# Synthesis and characterization of biopolyurethane crosslinked with castor oil-based hyperbranched polyols as polymeric solid–solid phase change materials

**DOI:** 10.1038/s41598-022-17390-x

**Published:** 2022-08-27

**Authors:** Joo Hyung Lee, Seong Hun Kim

**Affiliations:** 1 Department of Organic and Nano Engineering, Hanyang University, Seoul, 04763 Republic of Korea; 2 The Research Institute of Industrial Science, Hanyang University, Seoul, 04763 Republic of Korea

**Keywords:** Energy storage, Biopolymers, Polymer synthesis, Polymer characterization

## Abstract

Novel crosslinking bio polyurethane based polymeric solid–solid phase change materials (SSPCM) were synthesized using castor oil (CO) based hyperbranched polyols as crosslinkers. CO-based hyperbranched polyols were synthesized by grafting 1-mercaptoethanol or α–thioglycerol via a thiol-ene click reaction method (coded as COM and COT, respectively). Subsequently, the three SSPCMs were synthesized by a two-step prepolymer method. Polyethylene glycol was used as the phase change material in the SSPCMs, while the CO-based hyperbranched polyols and two types of diisocyanate (hexamethylene diisocyanate (HDI) and 4,4'-diphenylmethane diisocyanate) served as the molecular frameworks. Fourier transform infrared spectroscopy indicated the successful synthesis of the SSPCMs. The solid–solid transition of the prepared SSPCMs was confirmed by X-ray diffraction analysis and polarized optical microscopy. The thermal transition properties of the SSPCMs were analyzed by differential scanning microscopy. The isocyanate and crosslinker types had a significant influence on the phase transition properties. The SSPCM samples prepared using HDI and COT exhibited the highest phase transition enthalpy of 126.5 J/g. The thermal cycling test and thermogravimetric analysis revealed that SSPCMs exhibit outstanding thermal durability. Thus, the novel SSPCMs based on hyperbranched polyols have great potential for application as thermal energy storage materials.

## Introduction

The continuous increase in fuel prices and efforts toward achieving carbon neutrality in recent years has necessitated the development of sustainable energy sources^1,2^. From this perspective, thermal energy storage (TES) has become an important part of renewable energy systems^3–5^. To meet the growing demand for cheaper and more efficient TES, phase change materials (PCM) have recently gained attention for their applicability in the development of TES devices^6^. PCMs exhibit a large latent heat capacity and uniquely maintain a constant temperature during latent heat transfer, which make them suitable for thermal energy management in various applications such as solar energy storage^7–9^, solar desalination^10–12^, building materials^13–15^, and waste heat recovery^16–18^. In the past decade, studies have focused on the suitability of polyethylene glycol (PEG)^19^, paraffin waxes^20^, fatty acids^21^, inorganic salt hydrates^22^, and the eutectics of these compounds^23^ as PCMs. However, the development of TES materials using pristine PCMs is challenging, it requires special packaging technology to support the solid–liquid phase transition. Compared to the pristine PCM, solid–solid PCMs (SSPCM) are economical and convenient to produce, face no risk of leakage, and exhibit shape stability^24–26^. Thus, various methods have been studied to fabricate SSPCMs, such as grafting, crosslinking polymerization, and introduction of carbon materials, titanium oxide, black phosphorus, and their hybrids^27–35^.

Polyurethane (PU) is one of the most important polymeric materials that has been widely applied in industries such as automotive, furniture, thermal shielding materials, and sound insulating materials, because it is economical and convenient to produce^36–39^. PU is a polymer synthesized through the addition polymerization between a multi-functional alcohol and diisocyanate. As only few types of commercial diisocyanate, the polyols used play a critical role in determining the properties of PUs. Recently, numerous studies on PU-based SSPCMs have been reported in which PEG was used as the PCM material^40–42^. A PU-based SSPCM typically has a crosslinked structure, which limits the dissolution and flow of the PEG soft segment. Several researchers have developed improved polymeric SSPCMs using multifunctional polyol as a crosslinking agent^31,43–45^.

The use of castor oil (CO) as a bio-based polyol, which can replace conventional petrochemical polyols, has attracted considerable attention because of its biodegradability, abundance, nontoxic nature, cost competitiveness, and biodegradability^32,46^. CO is primarily composed of ricinoleic acid, with a double bond on C–9 and a hydroxyl group on C–12; therefore, it has been frequently applied in studies on bio PU (BPU). However, the applications of CO-based PU have been limited because of its low crosslinking density, which leads to low mechanical properties and low productivity. To overcome such drawbacks, many studies have conducted to facilitate the use of CO by epoxidation^47^, transesterification^48^, ozonolysis^49^, and thiol-ene click reactions^50,51^. Previously, we have reported the use of the thiol-ene click reaction for grafting 1-mercaptoethanol and α-thioglycerol onto CO^52,53^.

In this study, two types of CO-based hyperbranched polyols were prepared, which were subsequently used in the production of BPU-based SSPCMs. The structural properties of SSPCM were analyzed by Attenuated total reflection (ATR)-Fourier transform infrared (FTIR) spectroscopy, while X-ray diffraction (XRD) analysis and polarized optical microscopy (POM) were conducted to determine the degree of crystallinity. The phase change properties of the SSPCMs were thoroughly examined by differential scanning calorimetry (DSC). After the thermal cycling test, DSC and FT-IR analysis were performed again to verify the thermal reliability of the SSPCMs. Additionally, the thermal stability of the SSPCMs was verified by thermogravimetric analysis (TGA).

## Experimental

### Materials

CO was supplied from Yakuri Pure Chemical Co., Ltd. Mercaptoethanol, α-thioglycerol, 2, 2-dimethoxy-2-phenylacetophenone (DMPA), acetic anhydride, pyridine, and dibutyltin dilaurate (DBTDL) were purchased from Sigma-Aldrich (USA). PEG (MW 4000 g mol^-1^) was supplied from Kanto Chemical Co., Inc. Ethyl acetate (EA), *N, N*-dimethylformamide (DMF), anhydrous MgSO_4_ and NaCl were supplied from Daejung Chemical (Korea). 4, 4'-diphenylmethane diisocyanate (MDI) was purchased from Tokyo Chemical Industry Co., Ltd. Hexamethylene diisocyanate (HDI) was supplied from Wako Chemicals. All reagents were used as received without further purification.

### Preparation of CO-based hyperbranched polyols

**Scheme 1:**
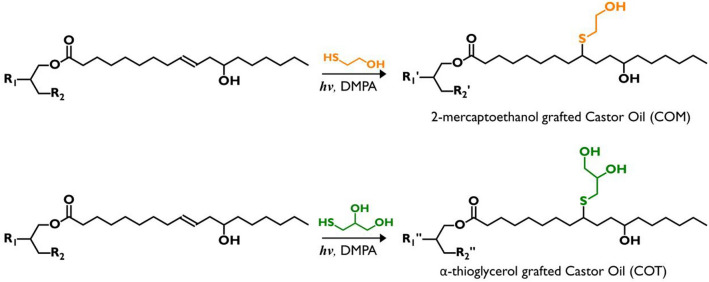
Preparation of CO-based multi-functional polyols via the thiol-ene coupling reaction.

The synthesis of CO-based hyperbranched polyols was carried out according to the method used in our previous studies^52,53^. The preparation scheme is shown in Scheme 1. CO, thiol, DMPA (as a photoinitiator), and EA (as a solvent) were placed in quartz tubes and rolled in a tube roller for 24 h after placing it in a photochemical reactor equipped with UV-A lamps. The molar ratio of the thiols to C=C double bonds of CO was set to 4:1. After the reaction, the products were washed with distilled water and aqueous NaCl solution at least five times. The obtained polyols were dried using MgSO_4_, and remained organic solvent was eliminated by rotary evaporation. The obtained polyols were then vacuum dried for 24 h. The CO‐based polyols grafted with 2-mercaptoethanol and α‐thioglycerol were coded as COM and COT, respectively.

### Preparation of SSPCMs

**Scheme 2:**
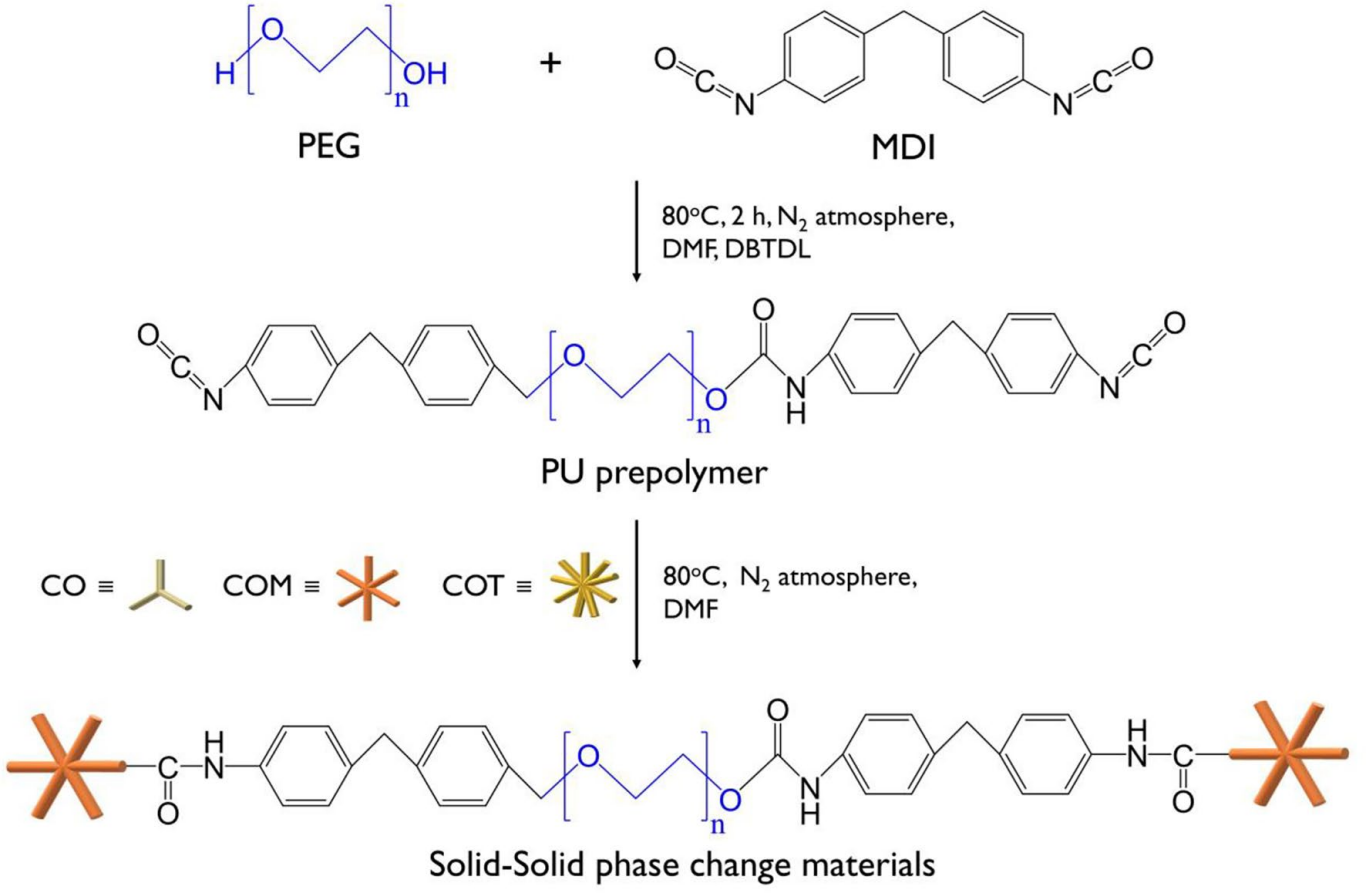
Pathway for the synthesis of the SSPCMs crosslinked with CO-based hyperbranched crosslinker.

As shown in Scheme 2, the synthesis of SSPCMs using PEG as a PCM was conducted using a two-step prepolymer method. First, a predetermined amount of PEG and the two types of diisocyanate (molar ratio of PEG and diisocyanate = 1:2) were separately dissolved in an appropriate amount of DMF, and the diisocyanate solution was added dropwise to the PEG solution with gentle stirring. A few drops of DBTDL (0.05 wt% based on the total weight of final product) were added as a catalyst. This reaction was carried out at 80 °C under N_2_ atmosphere for 3 h to obtain isocyanate-terminated prepolymers. Second, three types of crosslinkers were dissolved in DMF in adequate molar ratios and slowly added into prepared prepolymer solutions. After 18 h, the reaction mixtures were poured into a PTFE-coated mold and thermally cured at 80 °C for 24 h in a convection oven. The products were then kept *in vacuo* at room temperature for 24 h before analysis. The sample codes are listed in Table 1 according to the types of diisocyanates and crosslinkers used.

**Table 1:** Composition of the synthesized SSPCMs.

Sample code	Functionality ratio of PEG:diisocyanate:crosslinker	Type of crosslinker	Total NCO/OH ratio	PEG content (%)	*X*_*c, WAXD*_ (%)
M.CO	1:2.1:1	CO	1.05	76.1	72.6
M.COM	1:2.1:1	COM	1.05	81.1	76.3
M.COT	1:2.1:1	COT	1.05	83.1	77.7
H.CO	1:2.1:1	CO	1.05	78.7	77.9
H.COM	1:2.1:1	COM	1.05	84.1	78.0
H.COT	1:2.1:1	COT	1.05	86.0	78.4

### Characterization

ATR-FTIR spectrometer (Nicolet iS50, Thermo Fisher Scientific) was used to determine the structure of the SSPCMs over the range of 500–4000 cm^−1^. The FTIR spectra were recorded at a resolution of 4 cm^−1^, and 64 scans were averaged per experiment. Wide-angle XRD patterns were collected using an X-ray diffractometer (Rigaku, SmartLab), and diffractograms were scanned in a 2θ range of 5°‒70° at scan rate of 3°/min. The spherulite morphologies were observed through POM (Olympus BX-51 TX) equipped with a CCD camera. The thermal behavior of the SSPCMs was investigated by DSC (DSC 2010, TA Instrument). An accelerated thermal cycling test was performed for 100 cycles in the temperature range of 0‒100 °C using a thermocycler (MJ research). All samples were tested for 5 consecutive heating–cooling scan from 0 to 100 °C at 5 °C/min under nitrogen atmosphere. TGA of the SSPCMs was performed with nitrogen purging from 30 to 800 °C at 10 °C/min.

## Result and discussion

### Preparation of polyols

COM and COT were successfully synthesized under the optimized reaction conditions obtained from our previous studies^52,53^. The FTIR spectra of the newly prepared polyols are shown in Fig. S1. As stated in the supporting information, the chemical structures of the synthesized COM and COT are consistent with the results of our previous studies^52,53^. The hydroxyl values of CO, COM, and COT measured according to the ASTM D1957-86 standard were 160, 270, and 380 mg KOH g^−1^, respectively.

### Structural analysis of the SSPCMs

**Figure 1:**
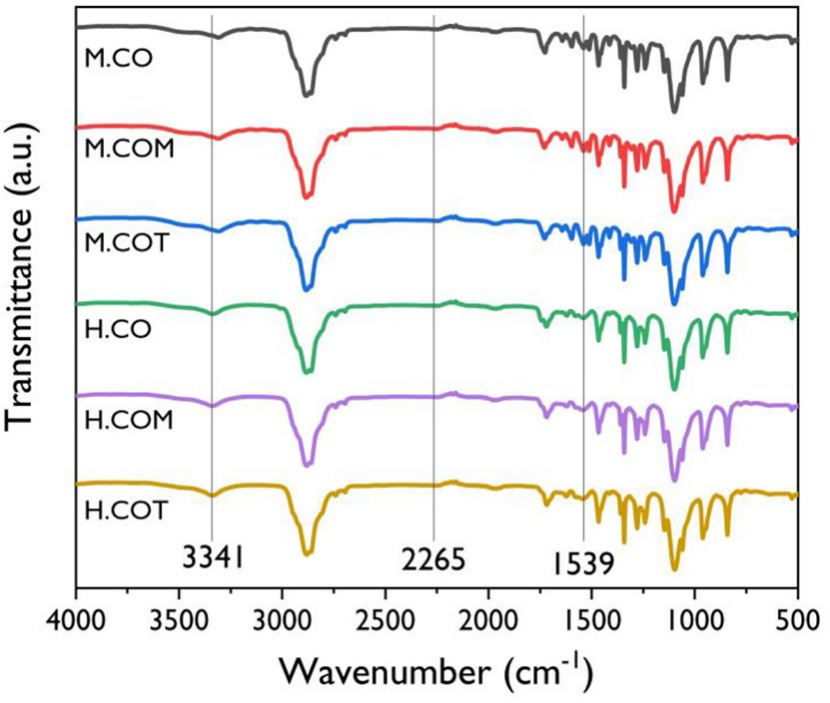
FT-IR spectra of the SSPCMs.

The chemical structures of the synthesized SSPCMs were analyzed by FTIR spectroscopy, and the results are presented in Fig. 1. The disappearance of the isocyanate peak at 2265 cm^−1^ in all the SSPCM spectra indicates that the PUs were successfully synthesized. The newly formed absorbance bands at 3341 and 1539 cm^−1^ correspond to the N–H stretching and amide vibration within the PU bond, respectively. The FTIR analysis did not show a significant difference in the spectra in terms of the functionality of the crosslinker or the type of isocyanate.

### Crystalline structure analysis of the SSPCMs

**Figure 2:**
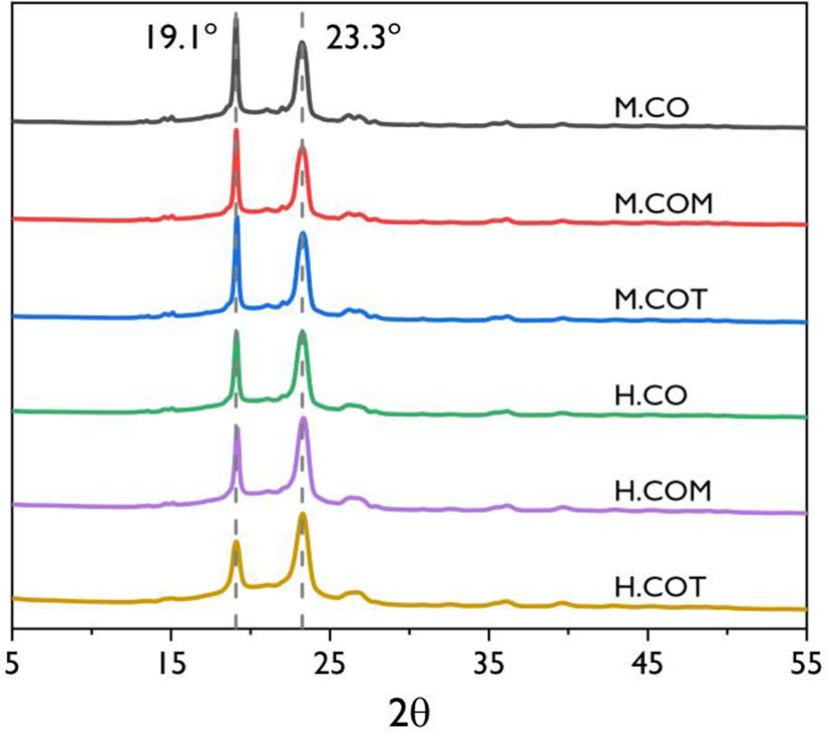
XRD patterns of the SSPCMs.

The crystal structures of the SSPCMs were analyzed by XRD analysis, and the results are presented in Fig. 2. Two strong diffraction peaks were detected in all the SSPCMs spectra, indicating that the SSPCMs had crystalline structures. PEG is known to exhibit sharp diffraction peaks at 19.1° (120) and 23.3° (032) implying that it is a semi-crystalline polymer with high crystallinity. The XRD patterns of the SSPCMs also showed peaks at 19.1° (120) and 23.3° (032), suggesting that the crystallinity of SSPCM is dominated by crystallinity of PEG. Compared to the XRD pattern of PEG, the SSPCMs had a lower peak intensity and a broader full width at half maximum. The crosslinked structure formed by hyperbranched polyols partially confined the PEG chain, which led to a decrease in the degree of crystallinity. Meanwhile, a significant difference in the degree of crystallinity was observed depending on the isocyanate type used. For PEG, the peak corresponding to the (032) plane in the XRD pattern had a higher intensity than that to the (120) plane. However, for the SSPCMs prepared using MDI, the intensity of the peak corresponding to the (032) plane decreased; for the SSPCMs prepared using HDI, the crystal structure of the pristine PEG was retained as the functionality of the crosslinker increased. The reduced intensity of the peak corresponding to the non-equatorial (032) plane of the SSPCMs prepared using MDI implied that thinner PEG lamellae and tilt of the PEG chain in the lamellae were generated with the formation of the crosslinked structures^54^. This was more prominent for the SSPCMs prepared using MDI because the rigid benzene ring further confines the PEG chain^41^.

**Figure 3:**
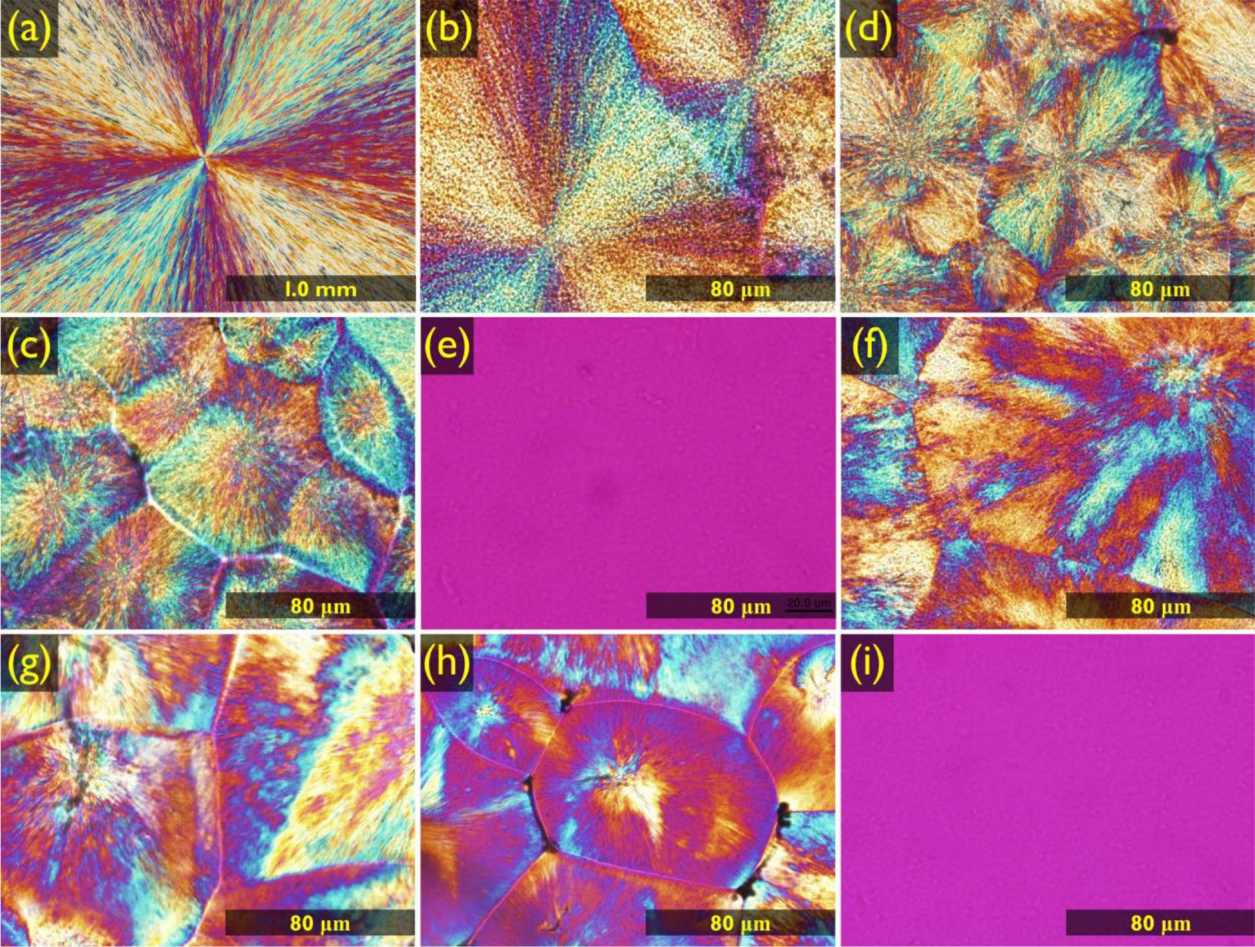
POM images of the (**a**) pristine PEG, (**b**) M.CO, (**c**) M.COM, (**d**) M.COT, (**e**) M.COT above 90 °C, (**f**) H.CO, (**g**) H.COM, (**h**) H.COT, and (**i**) H.COT above 90 °C.

**Figure 4:**
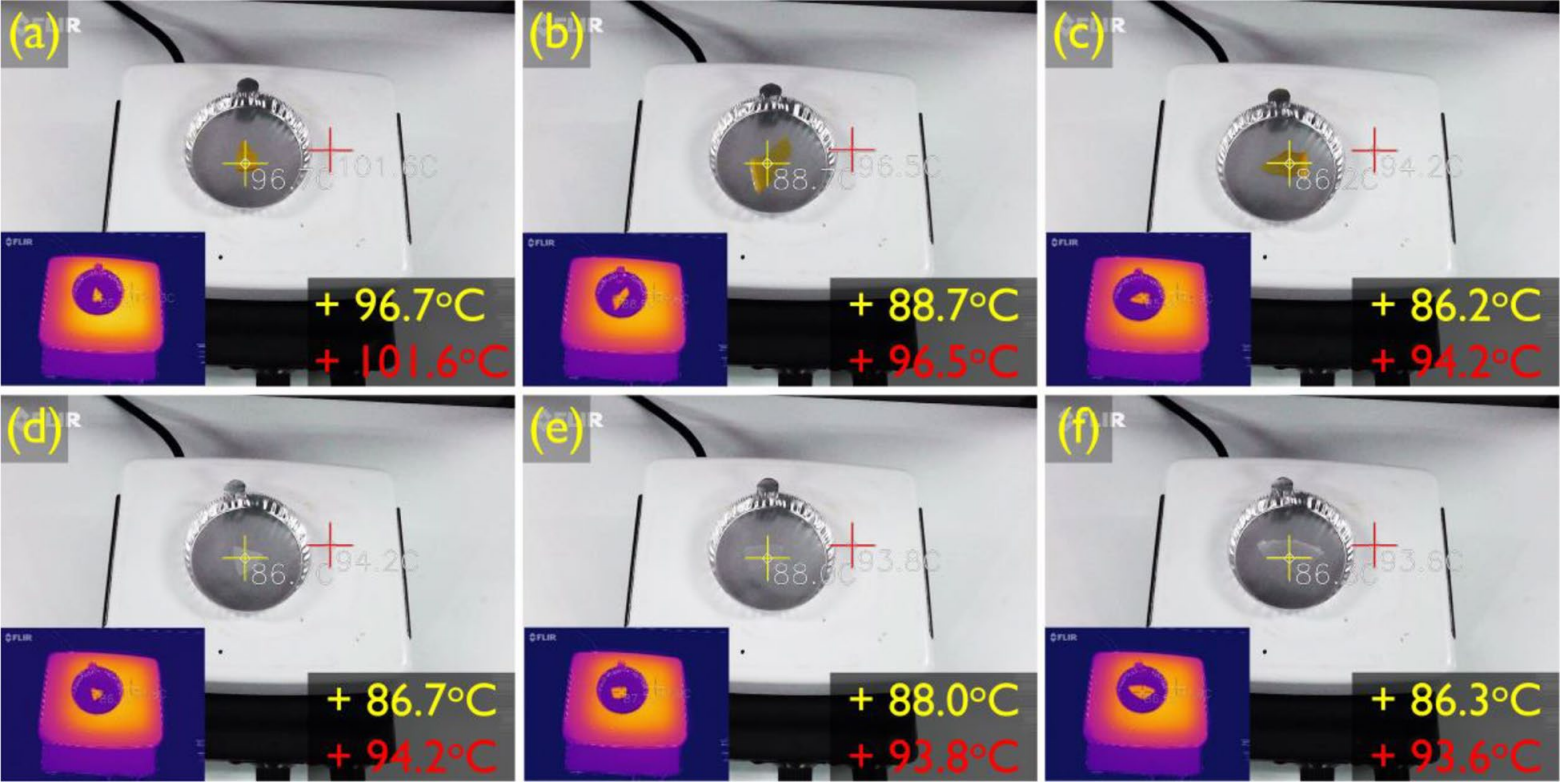
Images of the SSPCMs above 80 °C; (**a**) M.CO, (**b**) M.COM, (**c**) M.COT, (**d**) H.CO, (**e**) H.COM, and (**f**) H.COT.

For further analysis of the crystal structure of the synthesized SSPCMs, the crystal morphologies of the pristine PEG and the SSPCMs were recorded through POM, and the results are shown in Fig. 3. All SSPCM samples demonstrated cross-extinction patterns caused by polarized light. As shown in Fig. 3a, the crystal of pristine PEG grew to several millimeters in size, while the synthesized SSPCMs demonstrated a decrease in the crystal size because the crosslinked structure limited the motion of the soft segment. The crystal structure remained unchanged when the temperature of the SSPCMs was raised to the phase transition temperature. However, as the temperature reached the transition point, the crystal structure began to disappear and eventually no crystal features could be observed in the POM image (Fig. 3e and i). As shown in Fig. 4, the SSPCMs remained in the solid state even at a high temperature close to the phase transition temperature. The solid–liquid phase transition did not occur in all the synthesized SSPCMs, even at temperature higher than the transition point.

### Phase transition properties of the SSPCMs

DSC analysis was performed to examine the phase transition properties of the synthesized SSPCMs. The five consecutive DSC scans after heating and cooling of the pristine PEG and SSPCMs are presented in Figs. S2 and 5, respectively. For the pristine PEG, the solid–liquid phase transition resulted in very sharp exothermic and endothermic peaks in the range of 20–70 °C. The DSC scans for all SSPCMs also exhibited distinct exothermic and endothermic peaks, suggesting that the SSPCMs had reversible thermal storage and release properties. A consecutive heating–cooling test was performed to verify the potential reusability of the SSPCMs. The DSC thermograms remained unchanged during the test. The phase transition properties based on the five consecutive DSC scans are summarized in Table 2. The exothermic and endothermic enthalpies of all SSPCM structures were significantly lower than those of the pristine PEG. This implies that the heat storage and release capacities of the synthesized SSPCMs were lower than those of the pristine PEG. The solid–solid phase transition of the SSPCMs is due to the transformation of the PEG soft segment from a crystalline to an amorphous state. The PEG chains in the SSPCM structures were strongly confined to a finite interspace because of the crosslinked network formed by the hyperbranched crosslinker. Thus, the crystallization of the PEG chain was limited and certain PEG chains were prevented from crystallization in the phase transition process. Therefore, the crystal domain of the SSPCMs was reduced compared to that of the pristine PEG, resulting in reduced heat storage and release capacities. These inferences are supported by XRD analysis and POM results previously discussed in this study.

**Figure 5:**
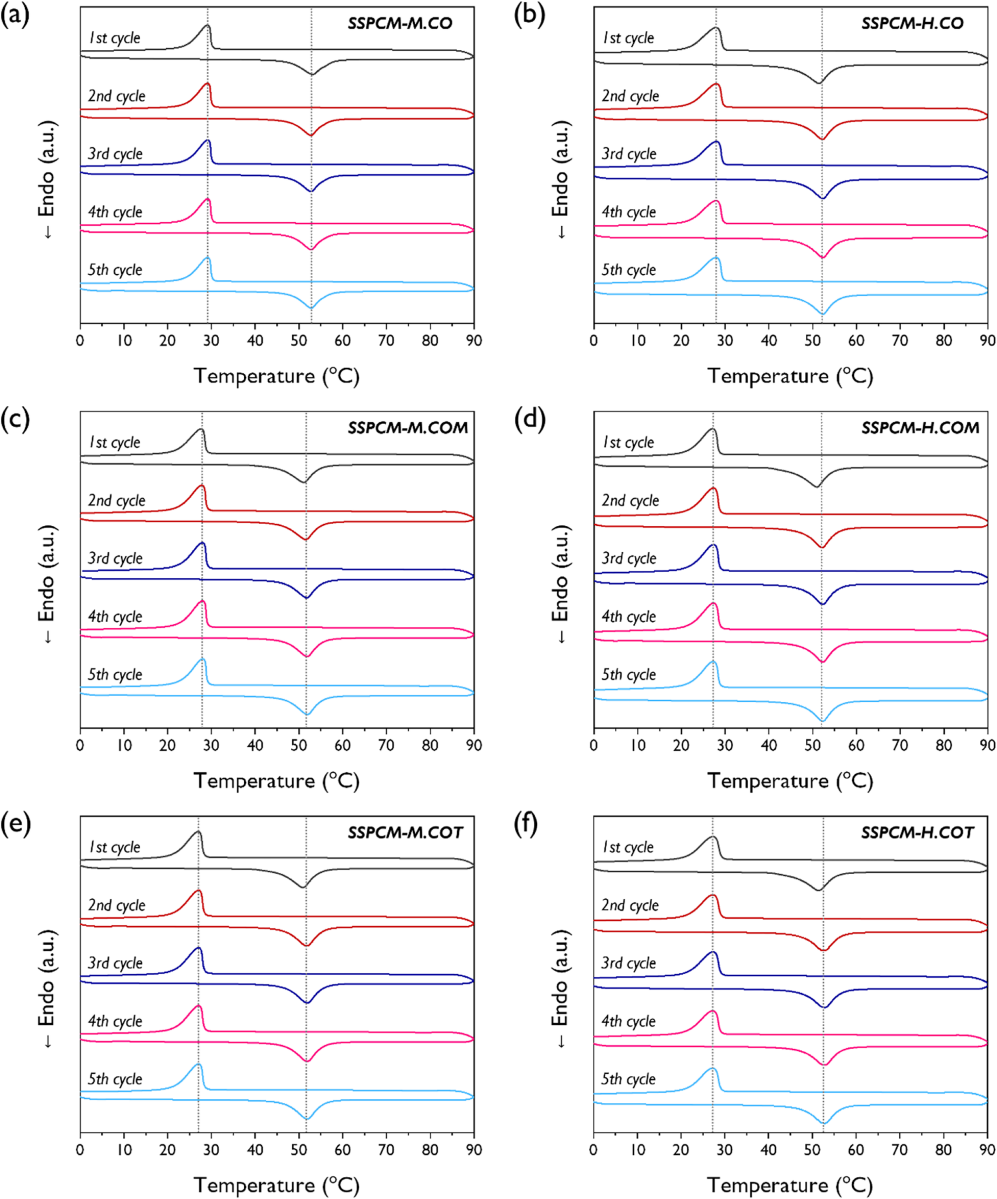
DSC curves of the SSPCMs with 5 consecutive heating–cooling cycles; (**a**) M.CO, (**b**) M.COM, (**c**) M.COT, (**d**) H.CO, (**e**) H.COM, and (**f**) H.COT.

**Table 2:** Phase-change characteristics of the SSPCMs calculated from 5 consecutive DSC scans.

Sample	Phase transition	Melting process		Freezing process		PEG content	Enthalpy ratio	Relative latent heat efficiency
		*ΔH*_*m*_ (J/g)	*T*_*m*_ (°C)	*ΔH*_*f*_ (J/g)	*T*_*f*_ (°C)	(%)	(R, %)	(η, %)
PEG 4000	Solid–liquid	206.5 ± 0.1	61.02 ± 0.03	180.7 ± 0.1	30.84 ± 0.01	–	–	–
M.CO	Solid–solid	110.6 ± 0.1	52.84 ± 0.07	109.4 ± 0.3	29.12 ± 0.01	76.1	53.5	70.4
M.COM	Solid–solid	115.7 ± 0.5	51.65 ± 0.16	112.4 ± 0.5	27.85 ± 0.08	81.1	56.0	69.1
M.COT	Solid–solid	117.9 ± 0.2	51.67 ± 0.20	114.8 ± 0.4	27.04 ± 0.02	83.1	57.1	68.8
H.CO	Solid–solid	117.8 ± 0.4	52.16 ± 0.17	115.2 ± 0.6	27.26 ± 0.04	78.7	57.1	72.5
H.COM	Solid–solid	124.5 ± 0.5	52.00 ± 0.26	119.8 ± 0.3	27.26 ± 0.04	84.1	60.3	71.8
H.COT	Solid–solid	126.5 ± 0.4	52.54 ± 0.29	122.0 ± 0.4	27.23 ± 0.01	86.0	61.2	71.2

Regarding the influence of the functionality of the crosslinker on the changes in the SSPCMs endothermic enthalpy, the degree of latent heat for both MDI- and HDI-based SSPCM followed the order; CO < COM < COT. Studies have revealed several factors that influence the SSPCM phase transition properties, including soft segment content by weight, crystalline state of the soft segments, and steric hindrance of the crosslinking points. With an increase in the functionality of the crosslinker, the synthesized SSPCM can accommodate more PEG, thereby increasing the latent heat storage capacity. For better understanding of the phase transition properties of the SSPCMs on the basis of the functionality of the crosslinker, the relative latent heat efficiency was calculated using the following equation:

$$\eta = \frac{{{\Delta }H_{m.SSPCM} }}{{{\Delta }H_{m.PEG} \times \omega_{PEG} }} \times 100$$

where ${\Delta }H_{m.SSPCM}$ and ${\Delta }H_{m.PEG}$ indicate the melting enthalpies of SSPCMs and the pristine PEG, respectively, and $\omega_{PEG}$ is the mass fraction of PEG in the SSPCM. Table 2 presents the relative latent heat efficiency of the SSPCM. The $\eta$ value indicates the influence of the framework structure, where higher values suggest less heat loss of the PEG in the SSPCM. Both MDI- and HDI-based SSPCMs exhibited $\eta$ values in the order of CO > COM > COT, suggesting that the increased level of steric hindrance caused by the increased crosslinking density led to an increase in the number of soft segments that cannot form the crystal structure during the phase transition process.

Considering the influence of the isocyanate type on the endothermic enthalpy of the SSPCMs, the latent heat of the HDI-based SSPCM was higher than that of the MDI-based SSPCM when an identical crosslinker was used. The $\eta$ value of H.CO, H.COM, and H.COT were shown to be increased by 3.0%, 3.8%, and 3.5%, respectively, compared to M.CO, M.COM, and M.COT, which is due to the presence of a rigid benzene ring in MDI that prevents PEG crystallization.

The analysis of the phase transition properties of the SSPCMs by DSC demonstrated that using hyperbranched polyols led to an increase in latent heat efficiency, although the relative latent heat efficiency decreased due to an increase in the crosslinking density. Steric hindrance arising from the structural characteristics of isocyanate is another factor influencing the latent heat efficiency of the SSPCMs.

**Figure 6:**
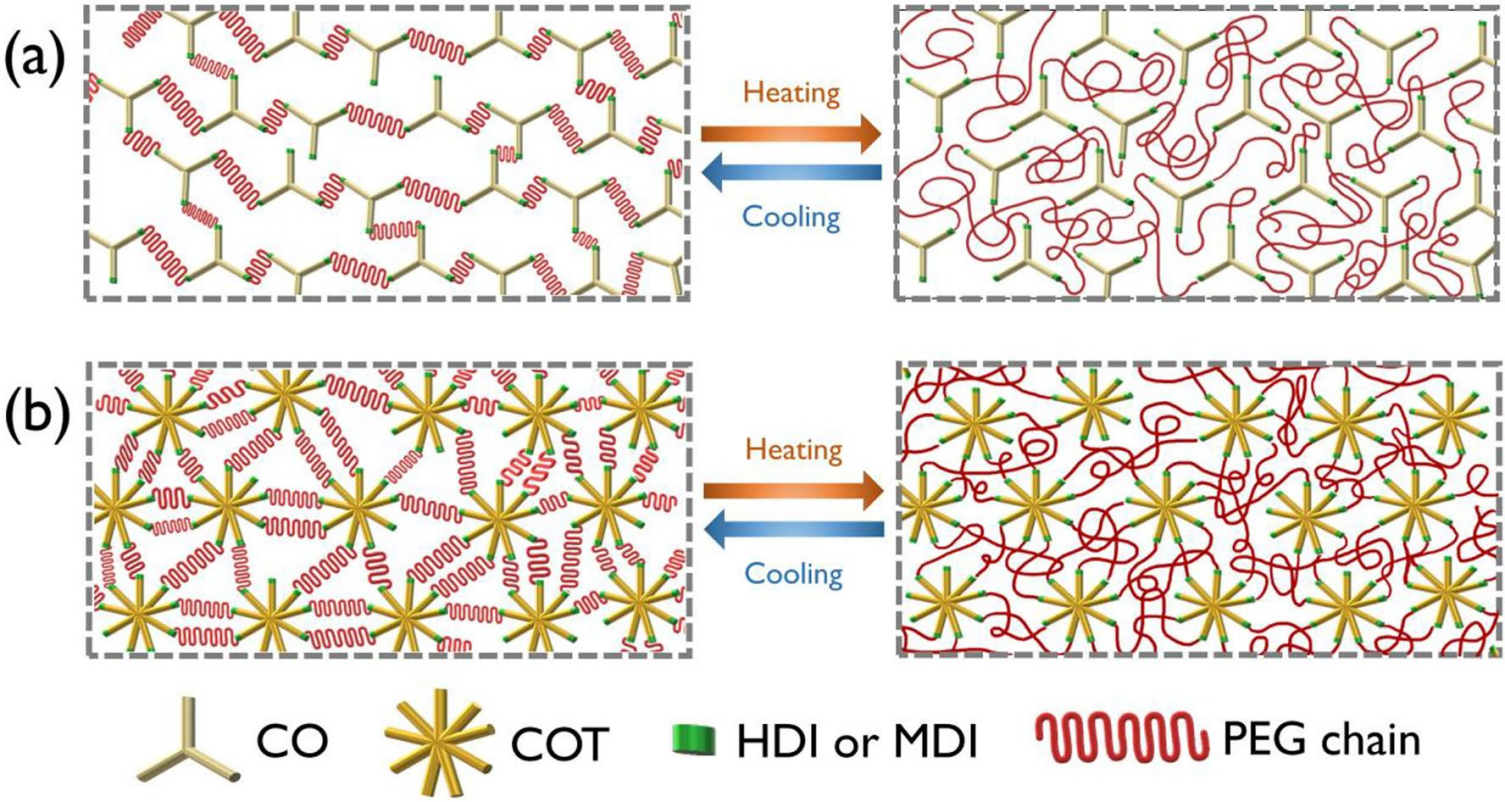
Schematic illustration of the phase change mechanisms of the SSPCMs crosslinked with (**a**) CO and (**b**) COT.

The results of the crystalline structure and phase transition property analyses confirmed that the synthesized SSPCMs exhibited repetitive heat storage and release characteristics without any leakage. The phase change mechanisms of the SSPCMs crosslinked with CO and COT are shown in Fig. 6. The crosslinked networks formed by the CO-based hyperbranched polyols served as the molecular framework, which prevented the dissolution and flow of the PEG soft segment. Compared with the CO-based SSPCM, the COT-based SSPCM exhibited higher thermal energy storage and release efficiencies because a greater amount of the PCM material can be integrated into it. Additionally, Table 3 listed the comparison of phase change properties of the H.COM and H.COT with those of other crosslinked polymeric SSPCMs in the literature. As clearly shown in the tabulated data, the latent heat capacity of the SSPCMs synthesized in present work is higher than the most of SSPCMs reported earlier ^40,41,55–64^.

**Table 3:** Comparison of thermal properties of the crosslinked polymeric SSPCMs in literatures.

SSPCMs	Heating rate (°C/min)	*T*_*m*_ (°C)	*ΔH*_*m*_ (J/g)	*T*_*f*_ (°C)	*ΔH*_*f*_ (J/g)	References
β-CD/MDI/PEG	2	60.2	115.20	47.8	111.6	^40^
PEG/MDI/Castor oil	10	51.4	117.7	42.3	109.0	^41^
PEG/PGMA copolymer	10	55.9	73.2	31.1	69.8	^55^
PEG/MDI/D-Sorbitol	2	59.7	107.5	44.0	102.9	^56^
Pentaerythritol/butane tetracarboxylic acid/PEG	–	53.21	102.8	17.83	100.1	^57^
Poly(acrylonitrile-co-itaconate)/PEG	–	53.05	96.95	34.15	97.46	^58^
P(mPEG5000A-DVB)	10	56.51	128.7	32.38	125.6	^59^
PEG/HMDI/Glycerol	5	45.0	91	20.2	89	^60^
PEG/PDMS/TEOS	10	62.99	124.5	25.77	104.4	^61^
PEG/HDIB/Graphene oxide	10	45.1	78.0	23.8	76.3	^62^
PEG/MDI/Xylitol	10	41.65	76.37	29.66	80.46	^63^
PEG/MDI/Xylitol/Lauric Acid	10	34.23	125.4	37.25	131.8	^64^
H.COM	5	52.00	124.5	27.26	119.8	Present study
H.COT	5	52.54	126.5	27.23	122.0	Present study

### Thermal reliability of SSPCM

To verify the thermal stability of the SSPCMs, an accelerated thermal cycling test (100 cycles from 0 to 100 °C) was performed. Even after the 100th thermal cycle, no weight reduction was observed in the SSPCMs, which indicates that they exhibit thermal reliability even after repeated use within the phase transition temperature range. This result verified the applicability of the SSPCMs as PCM materials.

**Figure 7:**
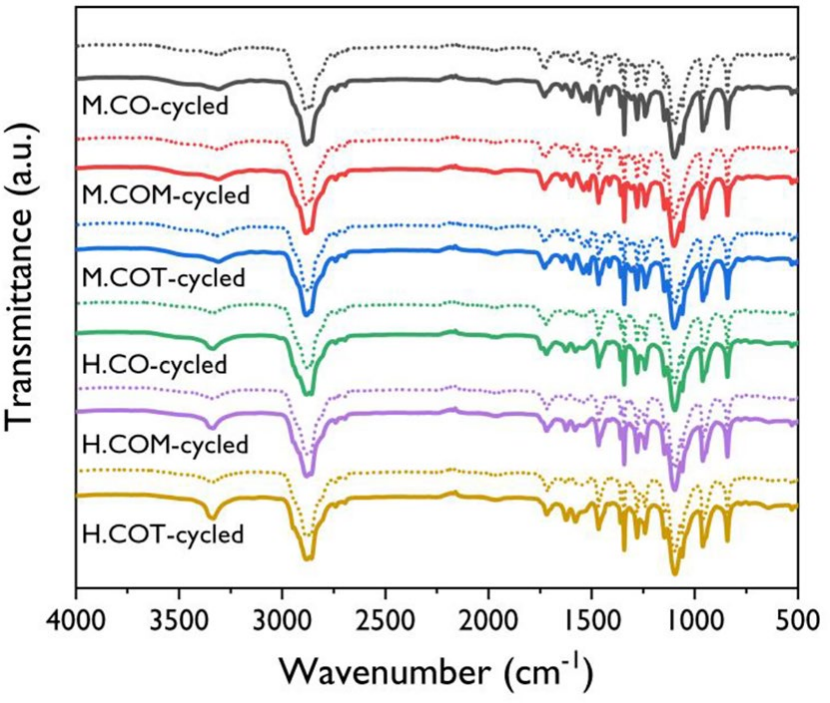
FT-IR spectra of the SSPCMs before (dot line) and after (solid line) the accelerated thermal cycling test.

Figure 7 shows the FTIR results for the SSPCMs after 100 cycles of the thermal cycling test. The initial FTIR curves for the SSPCMs are represented by dotted lines. Even after 100 cycles of the thermal cycling test, the FTIR curves for the SSPCMs displayed almost identical shapes and locations of the absorption peaks, suggesting that neither thermal decomposition nor structural changes occurred during repetitive thermal cycling.

**Figure 8:**
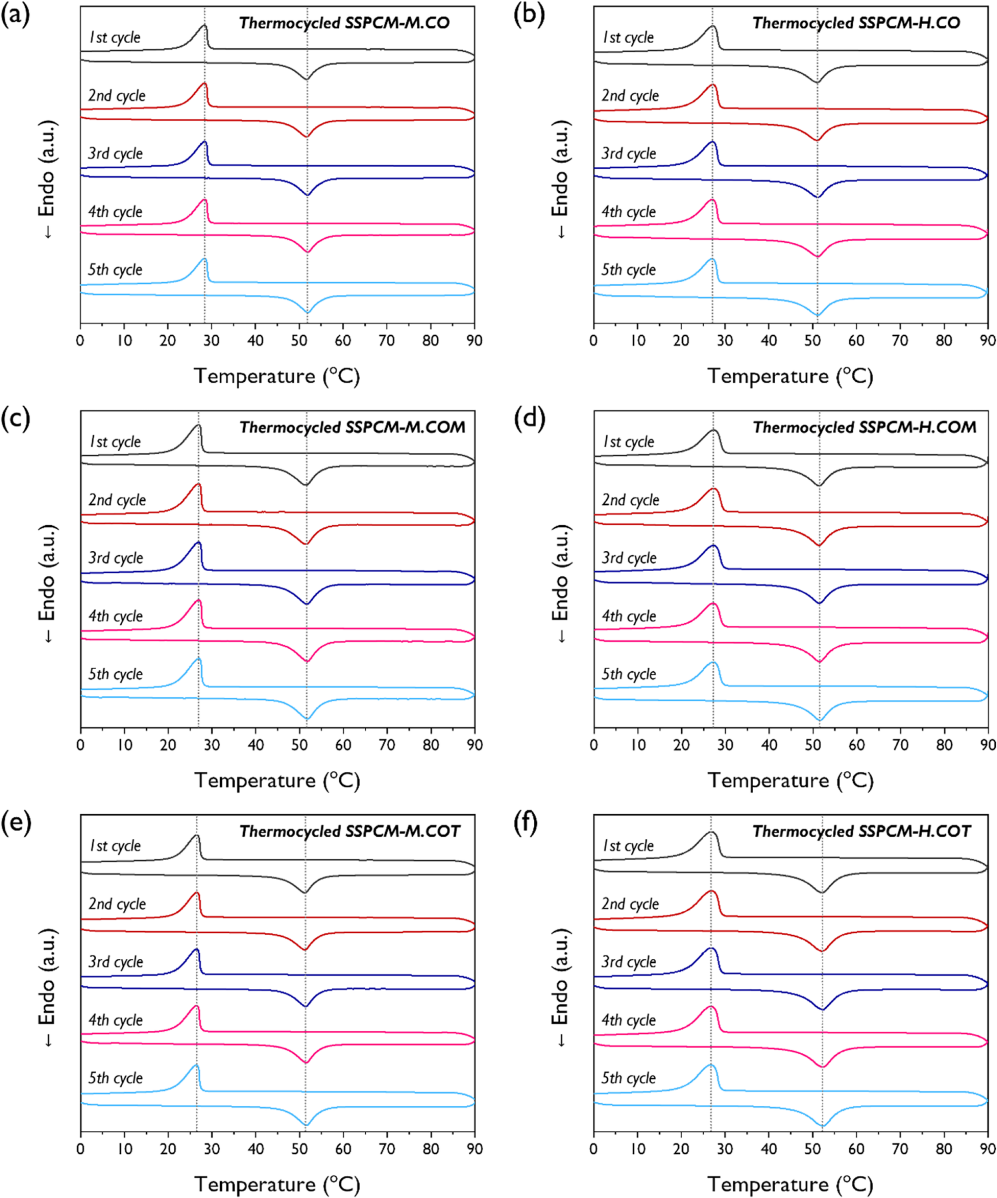
DSC curves of the SSPCMs with 5 consecutive heating–cooling cycles after the accelerated thermal cycling test; (**a**) M.CO, (**b**) M.COM, (**c**) M.COT, (**d**) H.CO, (**e**) H.COM, and (**f**) H.COT.

**Figure 9:**
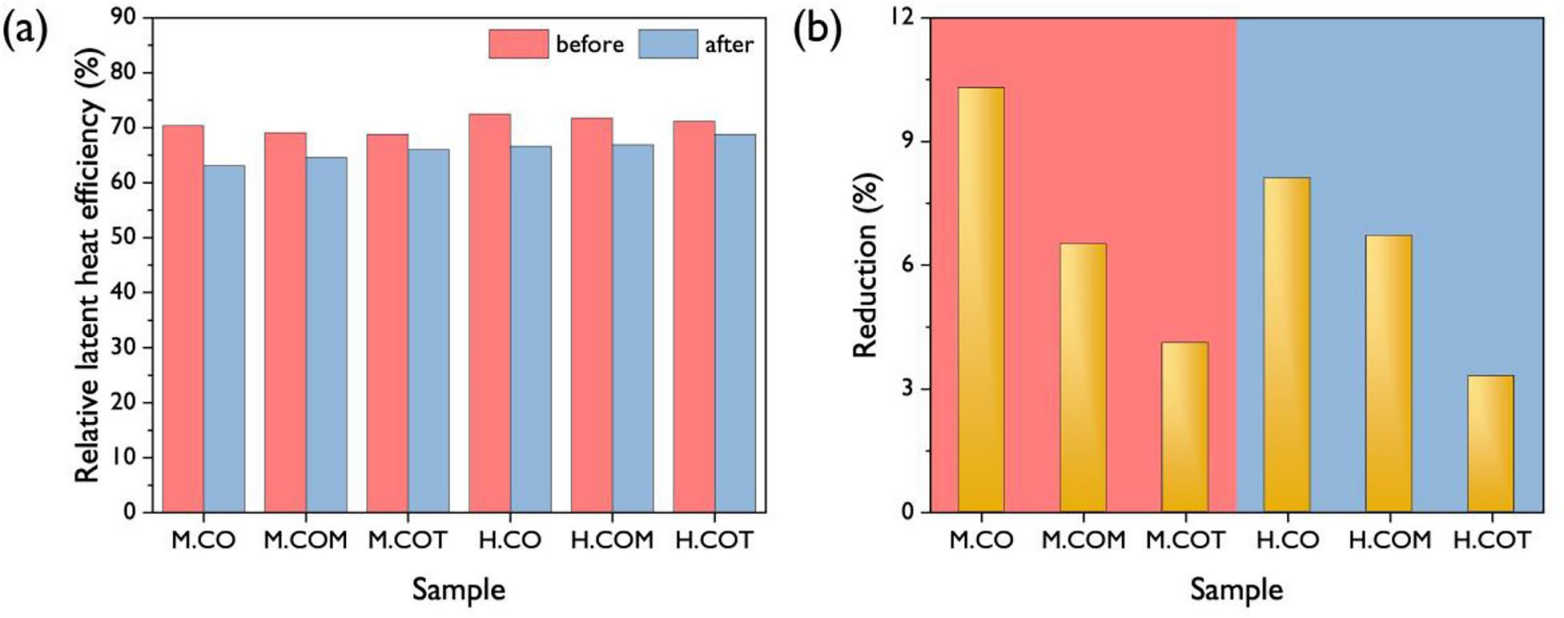
(**a**) Relative latent heat efficiencies of the SSPCMs before and after the accelerated thermal cycling tests and (**b**) the reduction in relative latent heat efficiencies.

Figure 8 shows the five consecutive DSC heating–cooling scans for the SSPCMs after the 100-cycle thermal cycling test; the phase transition characteristics are listed in Table 4. All the SSPCMs demonstrated distinct exothermic and endothermic peaks even after thermal cycling test and exhibited with a relatively uniform thermal behavior. These results confirmed that the SSPCM structures retained their reversible heat storage and release properties. However, the melting enthalpies decreased for all samples, and the degree of reduction of relative latent heat efficiency varied based on the isocyanate and crosslinker types. Figure 9 shows the initial relative latent heat efficiency of the SSPCMs followed by the final relative latent heat efficiency and the degree of reduction in the relative latent heat efficiency after thermal cycling. MDI-based SSPCMs exhibited a greater decrease in efficiency than HDI-based SSPCMs. Regardless of the isocyanate type, the reduction of efficiency gradually became smaller as the crosslinker functionality increased; M.COT and H.COT demonstrated reductions by 4.1% and 3.3%, respectively. Thus, hyperbranched polyols are feasible for use as crosslinkers for the production of highly efficient and durable SSPCMs.

**Table 4:** Phase-change characteristics for the SSPCMs calculated from 5 consecutive DSC scans after accelerated thermal cycling test.

Sample	Phase transition	Melting process		Freezing process		Enthalpy ratio	Relative latent heat efficiency	Reduction rate (%)
		*ΔH*_*m*_ (J/g)	*T*_*m*_ (°C)	*ΔH*_*f*_ (J/g)	*T*_*f*_ (°C)	(R, %)	(η, %)	
M.CO	Solid–solid	101.3 ± 0.4	51.84 ± 0.08	101.1 ± 0.5	28.38 ± 0.01	49.1	63.1	10.3
M.COM	Solid–solid	108.1 ± 0.8	51.62 ± 0.09	106.2 ± 0.5	26.92 ± 0.01	52.4	64.6	6.5
M.COT	Solid–solid	113.1 ± 1.1	51.36 ± 0.09	111.4 ± 0.5	26.47 ± 0.03	54.8	66.0	4.1
H.CO	Solid–solid	110.6 ± 0.7	51.12 ± 0.04	109.7 ± 1.2	27.10 ± 0.04	53.6	66.6	8.1
H.COM	Solid–solid	116.1 ± 0.6	51.46 ± .0.03	113.3 ± 0.7	27.23 ± 0.03	56.3	66.9	6.7
H.COT	Solid–solid	122.5 ± 0.4	52.26 ± 0.04	119.9 ± 0.1	26.80 ± 0.03	59.2	68.8	3.3

**Figure 10:**
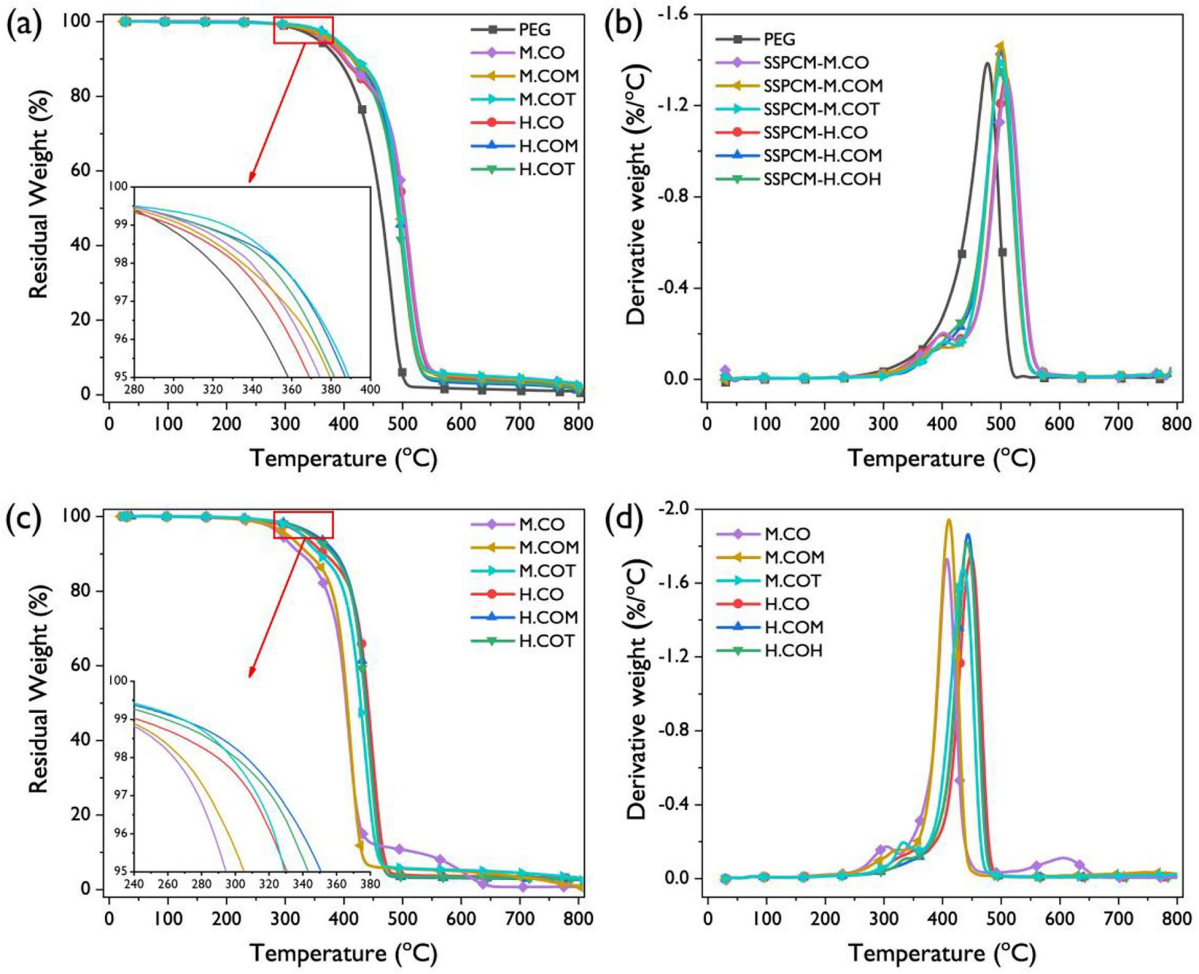
(**a**) TGA thermograms of the SSPCMs and (**b**) their derivates. (**c**) TGA thermograms of the SSPCMs and (**b**) their derivates before and after the accelerated thermal cycling tests.

TGA was performed to evaluate the thermal stability of the SSPCMs; the results are presented in Fig. 10. The weight of the pristine PEG and SSPCMs remained unchanged up to approximately 280 °C, implying that the SSPCMs were highly stable in the phase transition temperature range, facilitating TES application. Moreover, all the SSPCMs exhibited thermal decomposition at temperatures higher than those for the pristine PEG, which can be attributed to the crosslinked molecular structure of the SSPCMs. The derivative TGA curves in Fig. 10b shows that the SSPCMs exhibited two phases degradation behavior in contrast to the phase 1 thermal degradation behavior of the pristine PEG. Phase 1 is marked by the thermal degradation of the urethane bonds in the SSPCMs, whereas phase 2 is represented by the degradation of PEG^37,65^. Also, the maximum decomposition temperature of all the SSPCMs was higher than that of PEG, exhibiting the outstanding thermal stability of the SSPCMs.

## Conclusion

In this study, two types of hyperbranched polyols, COM and COT, with an increased hydroxyl value, were prepared via the thiol-ene click reaction with CO. A new SSPCM series for TES was successfully prepared using PEG as the PCM in the SSPCMs, while CO, COM, and COT provided the molecular framework. FTIR analysis of the SSPCMs revealed the formation of PU structures, which confirmed the successful synthesis of the SSPCMs. Moreover, the results of the XRD analysis and POM indicated the solid–solid phase transition. The results of the DSC analysis revealed that the isocyanate and crosslinker types had a significant influence on the phase transition properties. The H.COT exhibited the highest phase transition enthalpy at 126.5 J/g. Furthermore, the results of the thermal cycling test and TGA demonstrated the outstanding durability of the SSPCMs. Thus, the novel SSPCMs based on hyperbranched polyols has great potential to be applied in TES materials.

## Supplementary Information

Supplementary Information.
