# Supplementary material for: Synthesis and characterization of biopolyurethane crosslinked with castor oil-based hyperbranched polyols as polymeric solid–solid phase change materials
Source: Sci Rep. 2022 Aug 27;12:14646. doi: 10.1038/s41598-022-17390-x (PMC9420148; doi:10.1038/s41598-022-17390-x)
Supplement: Supplementary file 1 — Supplementary Information. [file 41598_2022_17390_MOESM1_ESM.docx]

**SUPPORTING INFORMATION**

**Synthesis and characterization of biopolyurethane crosslinked with castor oil-based hyperbranched polyols as polymeric solid-solid phase change materials**

Joo Hyung Lee^1, 2^ and Seong Hun Kim^1,^ *

*^1^Department of Organic and Nano Engineering, Hanyang University, 222 Wangsimni-ro, Seongdong-gu, Seoul, Republic of Korea*

*^2^Research Institute of Industrial Science, Hanyang University, Seoul, Korea*

*Corresponding Author: Prof. Seong Hun Kim, Ph.D. (E-mail: [kimsh@hanyang.ac.kr](mailto:kimsh@hanyang.ac.kr))


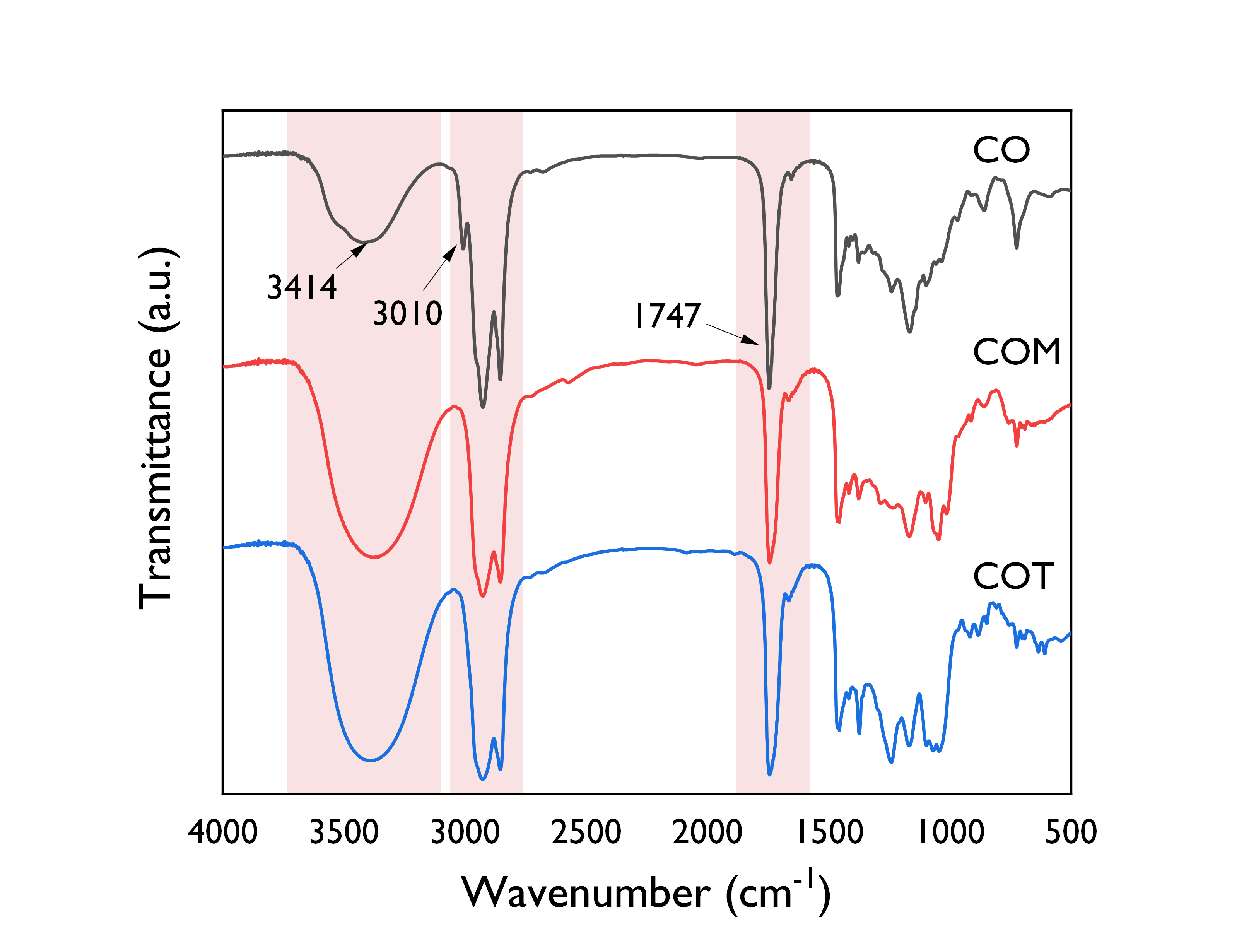


**Figure S1.** FT-IR spectra of CO, COM, and COT.

The structure of the prepared polyols was investigated by Fourier transform infrared spectroscopy. The bands at 1747cm^-1^, which is correspond to the carbonyl stretching vibration, were used for normalization. Grafting of the thiols onto castor oil resulted in the disappearance of the C=C–H absorption band at 3010 cm^-1^. Broad bands at 3414 cm^-1^ corresponding to the hydroxyl groups increased with an increase in functionality. These observation is consistent with our previously reported results, and it can be confirmed that the synthesis of COM and COT was confirmed to be successful ^1,2^.


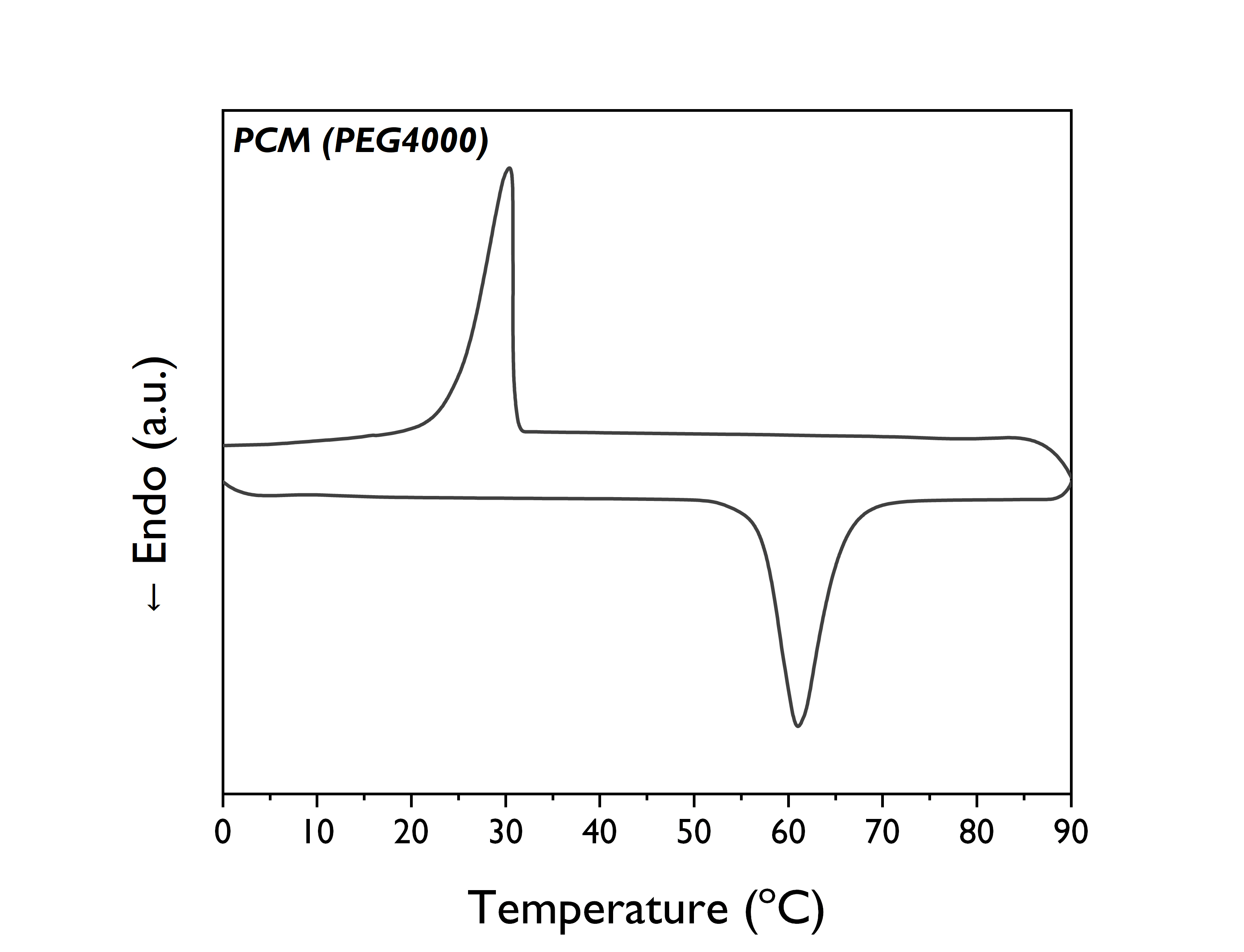


**Figure S2.** DSC curve of the pristine PEG.

**References**

1 Park, C. K., Lee, J. H., Kim, I. S. & Kim, S. H. Castor oil‐based polyols with gradually increasing functionalities for biopolyurethane synthesis. *J Appl Polym Sci*, 48304 (2019).

2 Lee, J. H., Kim, S. H. & Oh, K. W. Bio-based polyurethane foams with castor oil based multifunctional polyols for improved compressive properties. *Polymers-Basel* **13**, 576 (2021).
